# Emerging and re-emerging viruses affecting the nervous system

**DOI:** 10.1186/s42466-019-0020-6

**Published:** 2019-06-11

**Authors:** Uta Meyding-Lamadé, Eva Craemer, Paul Schnitzler

**Affiliations:** 1 Department of Neurology, Krankenhaus Nordwest GmbH, Steinbacher Hohl 2-26, 60488 Frankfurt/Main, Germany; 2 Zentrum für Infektiologie, Virologie, Universitätsklinikum Heidelberg, Im Neuenheimer Feld 324, 69120 Heidelberg, Germany

**Keywords:** Emerging viruses, Dengue-virus, Japanese-encephalitis-B-virus, Meningoencephalitis, Neuroinfectious disease, Nipahvirusencephalitis, Vector-borne disease

## Abstract

Emerging and re-emerging viruses may cause meningitis, encephalitis, meningoencephalomyelitis, encephalitis, Guillian-Barré-like-syndromes as well as strokes. Most important viruses belong to the family of *Adenoviridae, Arbovirus, Arenaviridae, Herpesviridae, Picornaviridae, Paramyxoviridae* as well as *Togaviridae*.

Clinical presentation usually consists of a biphasic presentation. Non-specific febrile illnesses may be accompanied by rash, headache, arthralgia and myalgia. Thereafter focal neurological signs may evolve. Diagnostic strategies for the detection of emerging and re-emerging viruses may be difficult due to the short viraemic period. Pitfalls in serology may be due to antibody crossreactivity.

Arboviruses are transmitted by arthropods. Aedes mosquitos are one of the vectors for arboviruses like Chikungunya-virus, Dengue-virus, Japanese-Encephalitis-B-virus and West-Nile-virus. Since the last centuries Aedes mosquitos have spread from their naturally habitat in Africa to America as well as Europe. The arboviruses risk profile depend essentially on the occurrence, the activity of the respective vector, this may be the key to fight the disease and its spread. Due to global shifts in the ecological balance but also as a result of more or less successful control measures, some diseases have become rarer, others are more common. The viruses persist in the respective vector months to years; in ticks they may persist for years and in mosquitoes 1 to 4 months. In order to survive bad climatic conditions unscathed, the viruses partially overwinter in arthropods.

## Introduction

The increased trade, travel habits, urbanization and climate change are some of the factors, which favour evolution and spread of new pathogens. Infectious diseases are emerging or re-emerging every year. Neuroinfectious diseases may occur as outbreaks in small localized regions or may spread rapidly over large geographical areas. An epidemic occurs when an infectious disease spreads rapidly to many people like the Zika Virus outbreak. A pandemic is a global disease outbreak as seen e.g. in HIV/AIDS. Recent outbreaks caused by Chikungunya-virus, West – Nile-virus, Enterovirus infection and Ebolavirus have caused severe neurological manifestations and spread rapidly across continents. Endemic diseases are constantly present, usually in low numbers. Neuroinfectious diseases transmitted by viruses may cause a broad spectrum of neurological presentations such as meningitis, encephalitis, meningoencephalitis, Guillian-Barré-like-syndromes as well as strokes. Often these patients are left with severe neurological sequelae. It is obligatory that neurologists are aware of current developments including typical and atypical presentations of neurological infections in travellers, diagnostic and treatment recommendations. To avoid fatal outcomes and long-term sequelae, specialized neurological intensive care medicine is crucial for survival.

Neurotropic viruses can access the brain via retrograde axonal transport, haematogenous spread crossing the blood-brain-barrier (BBB) or through spread of infected leukocytes across the BBB into the brain parenchyma. In the pathogenesis of neurotropic pathogens the blood brain barrier plays a vital role by controlling the access of circulation molecules, immune cells or viruses into the central nervous system. The BBB is not impenetrable and neurotropic viruses have evolved to disrupt and evade. But it’s known that some of the viruses are able to invade the brain parenchyma like a “Trojan horse”: retrograde axonal transport of a Flavivirus after a mosquito bite provides such a neuroinvasive pathway. Semaphorins (secreted, transmembrane and glycosylphosphatidylinositol-linked proteins) act as a potential entry receptor. Viral infection increases metalloproteinase and tumour necrosis factor levels such facilitating entry of infected neutrophils into the CNS. Flaviviruses like West-Nile-virus, Dengue-virus, Japanese-Encephalitis-B -virus may use this route to invade the CNS. Paracellular entry of virus includes alteration in expression or phosphorylation of tight junction proteins, disruption of the basal lamina and disruption of the actin cytoskeleton.

Many viruses causing neurological disorders belong to the family of *Adenoviridae, Arboviruses* (arthropod-borne-virus)*, Arenaviridae, Herpesviridae, Picornaviridae, Paramyxoviridae* as well as *Togaviridae*. Since the last few years also *Bornaviridae* (Bornavirus) have been discussed as possibly associated with psychiatric disorders. In 2015 a new lineage (variegated squirrel 1 bornavirus, VSBV-1) was confirmed as causative pathogen for encephalitis in humans.

Arboviruses are transmitted by arthropods, about 100 of them are human pathogens, which represent a significant public health problem throughout most parts of the world. The term arbovirus comprises many different genetically inhomogeneous viruses being only defined by the transmission through arthropods.

Arboviruses multiply in vertebrates, are picked up by arthropods such as blood-sucking insects or ticks during viremia and transmitted to vertebrates by bite or string. In order to survive bad climatic conditions, the viruses partially overwinter in arthropods. With a few exceptions humans and pets like cats and dogs are no hosts for arboviral infection [6].

Flavivirus, family *Flaviviridae*, belongs to the most important developing viruses worldwide and comprises over 70 different virus types. Flaviviruses utilize a single-stranded mRNA and translate into precursor proteins, which includes metalloproteinases, structural as well as non-structural proteins.

Flaviviruses use mosquitos like *Aedes spp.*, Culex mosquitos or ticks (*Ixodidae, Argasidae*) as vectors. Insect-borne viruses usually have specific endemic geographical areas where the incidences are mostly related to the seasons. Flaviviruses are present in six different continents and global distribution of flaviviruses is dynamic [17].

**Fig. 1:**
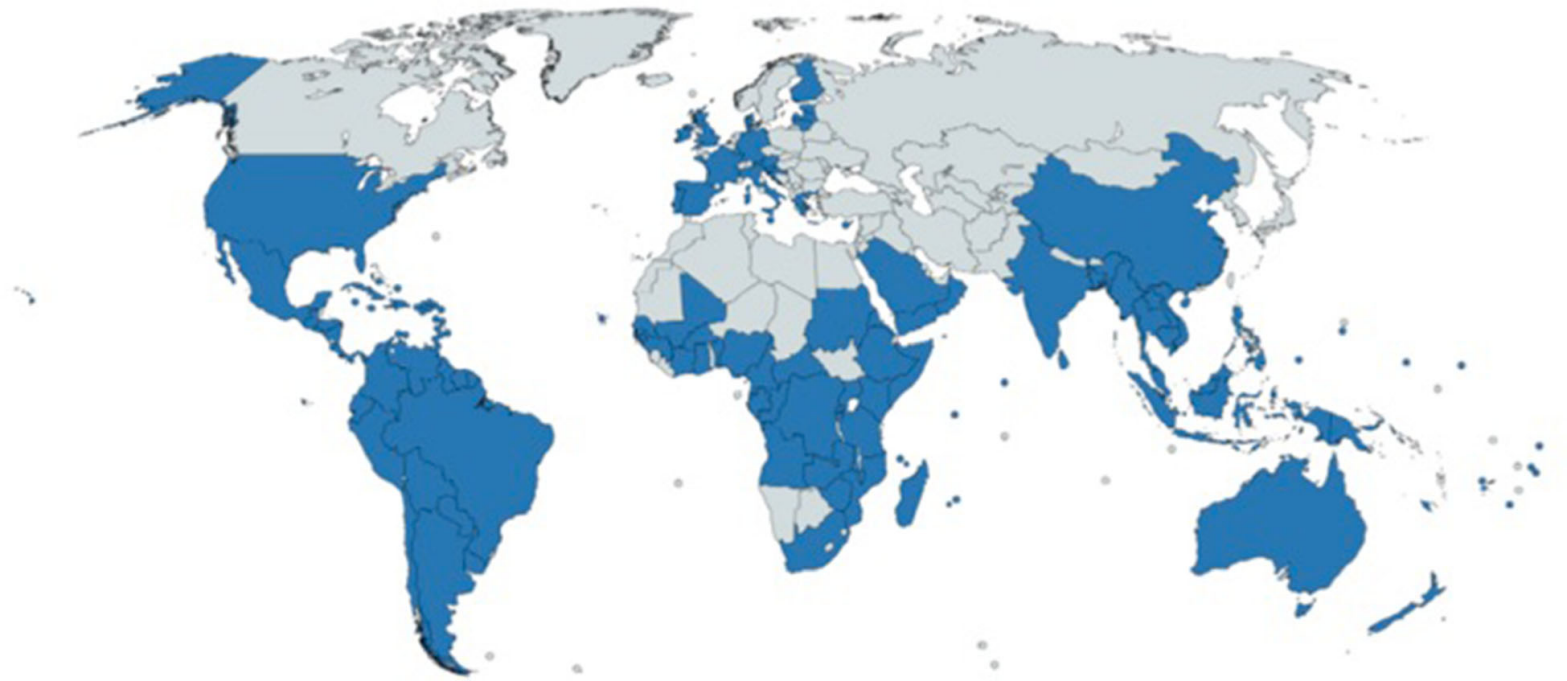
Distribution of Aedes mosquitos (created with mapchart.net 27.10.2018)

Dengue-virus (DENV), Zika virus (ZIKV), Chikungunya-virus, Japanese Encephalitis, tick-borne encephalitis, West-Nile-virus and St. Louis Encephalitis are among the most common flaviviral infections in which the CNS is involved. The Asian tiger mosquito is an important transmitter of Arboviruses, the yellow fever mosquito (*Aedes aegypti*). Among the more than 22 types of viruses that can be transmitted by *Aedes albopictus* are Chikungunya, Dengue, Yellow Fever and West-Nile-viruses. Figure 1 shows the current distribution of Aedes mosquitos, originally Aedes mosquitos are first recognized in Africa, spreading to southeast Asia and during the last decades, mostly all over the world.

The name flavivirus is derived from the first virus (yellow fever), for which an insect-bound pathway was detected (flavus, Latin: yellow) [24, 25].

Due to the close degree of relationship as well as co-circulating in the same geographical regions coinfections with several arboviruses can occur. Cases of Chikungunya virus (CHIKV) and Dengue-Virus (DENV) co-infections as well as Zika virus (ZIKV) and CHIKV co-infections have been reported [14, 23, 27].

However, it’s unclear whether the co-infection occurs via single or multiple mosquito bites.

Goertz et al. have investigated the potential of *Ae. aegypti* to transmit both ZIKV and CHIKV in one bite. In their results 12% of mosquitos transmit ZIKV and CHIKV in one bite, the simultaneous oral exposure to both viruses didn’t change infection and transmission rate compared to exposure to single virus. [10]. Some cases with triple infection with ZIKV,CHIKV and DENV have also been reported (Waggoner et al.; [4, 26]).

Viral infections may cause direct virus mediated cell damage as well as autoimmune mediated disorders. Another mechanism is the so-called immune enhancement - after primary infection of one subtype of Dengue-virus and reinfection with another Dengue-virus subtype causes an aggravation of symptoms and severe neurological manifestation [20, 21].

### Dengue-virus (DENV) infection of nervous system

#### Taxonomy, species, morphology, genome, reproduction, pathogenesis, virulence, transmission

Dengue is a member of the family *Flaviviridae* and has a single-stranded positive RNA, with four known serotypes, DENV-1 to DENV-4. DENV is transmitted by Aedes spp. (*Aedes aegypti, Aedes albopictus*), nevertheless not every Aedes spp. transmits every serotype due to their genetic variations.

Mosquitos without the receptor protein R67 / R64 in their gut epithelial cells are not capable to transmit DENV 1–4. Through the bite of the infected mosquito, the DENV enters the bloodstream and invades the macrophages. The macrophages transport the virus via the lymph channels to the lymph nodes, where it replicates. The viremic patient has between 10^8^ and 10^9^ infectious particles per ml of blood, and the viremia lasts four to 5 days on average. Endothelial cells and possibly also bone marrow cells are susceptible to infection. A previous infection with DENV 1–4 followed by another of the four DENV serotypes can lead to more serious illnesses, which maybe explained by immunoenhancement [20].

Comparing the amino acid sequences of their E proteins of the four DENV serotypes, they show 63–68% homology between the different serotypes. The homology between different variants of a DENV serotype is over 90%. Partially, the DENV-specific antibodies cross-react with other serotypes in DHF and DSS. Due to their low affinity, they do not have a neutralizing effect, but confer a preferential, more efficient uptake into the cells of the viruses complexed with the AK through interaction with Fc receptors on monocytes and macrophages (the cross-reacting IgG molecules increase the infection). Osman et al. have conducted a study on the frequency of serotypes in DENV infection in Brunei Darussalam, Southeast Asia. The predominant serotype for Brunei is DENV-2, followed by DENV-1. [22], patients travelling for example to Malaysia and get infected with another DENV are expected for more severe diseases, most likely a phenomenon called immunoenhancement [3].

#### Epidemiology

In 1992, dengue fever (DF) was still considered by the World Health Organization (WHO) as one of the neglected diseases. Since then, a dramatic change has taken place: the disease is now regarded as one of the most important infectious diseases with pandemic proportions. The overall incidence has increased more than 30 times over the past 50 years. For example in Germany, 957 cases were reported in 2016 and 635 cases in 2017 to the Robert Koch Institute (RKI). In 2018, 367 cases have been submitted to the RKI up to the month of October. The annual number of cases is estimated at around 390 million worldwide. Dengue is endemic in more than 100 countries in Africa, America, Eastern Mediterranean, South-East Asia and Western Pacific regions. Most seriously affected are the Americas, South-East Asia and the Western Pacific regions.

#### Clinical manifestation

The incubation time ranges from 3 to 14 days. Dengue infections are usually clinically mild or even often asymptomatic. The neurological complications include meningitis and encephalitis. Patients initially present with fever, headache, muscle and joint pain and rash. Usually there is a sudden increase in fever. Headache, severe muscle and joint pain, bradycardia, hypotension, lymphadenopathy frequently occur, named breakbone-fever. It may come to a second fever peak with a body exanthema as well as petechiae. In general, the course of the disease is short and abrupt, but long periods of reconvalescence with severe exhaustion are described, which can last for weeks. Patients with concomitant hepatitis with moderate transaminase elevation regularly appear. In about 10% there is an involvement of the nervous system. The most common neurological presentations of DENV infection are encephalitis, meningitis, strokes and cerebellar haemorrhage, Guillain-Barré-Syndrome, myositis can also be caused by DENV.

Especially in this group, a pronounced fatigue for several weeks in the sense of a depletion syndrome often appears after expiration of the acute illness. The increased permeability of blood capillary walls as well as thrombocytopenia is the cause of dengue haemorrhagic fever (DHF) and dengue shock syndrome (DSS). The DHF has primarily similar symptoms to the DF, followed by a short remission. Before a sudden worsening characterized by hypotension and circulatory collapse, stomachache, vomiting, restlessness, impaired consciousness or a sudden change from fever to hypothermia are the progenitor of DHF. Petechiae in skin and mucous membranes occur secondary to low platelet count and dysfunctional thrombocytes. Intracranial haemorrhage, haemorrhagic pneumonia and gastrointestinal bleeding may occur. The DSS follows an unfavourable course of DHF with mostly lethal outcome. DHF is triggered by host factors such as a previous DENV infection and an age < 15 years. DHF and DSS are typical secondary diseases after a previous primary infection, through which different serotypes bind the originally dengue-targeted antibodies to the surface of the new serotype without being able to neutralize it. The virus is “masked” for another immune response, propagation and proliferation is favoured, this can lead to a serious disease course with bleeding tendency and vascular damage. DENV has also been detected in organs such as the lungs, liver, kidneys and gastrointestinal tract.

#### Differential diagnosis

Zika Virus Infection, Chikungunya virus fever, West-nile-fever, as well as other arthropod-borne viruses and Malaria or Leptospirosis, Guillain-Barré-Syndrome, Stroke [8].

#### Diagnosis

CSF examination is crucial for diagnosis here ELISA is the gold standard, rt-PCR may also be performed. Typically patients show thrombocytopenia. An increase in transaminases and lymphocytosis may occur. CSF has to be repeated in cases of doubt, if possible due to thrombocytopenia. The trias of rash, thrombocytopenia and severe headaches, muscle and joint pains is characteristics for the diagnosis of Dengue fever.

#### Treatment

There is no antiviral therapy available. DENV infection is treated symptomatically. Early recognition of severe complications for example secondary to thrombocyte dysfunction and thrombocytopenia is highly important. Treatment on a specialized neurological intensive care unit is recommendable, if myelitis or meningoencephalitis occurs neurologic intensive care is life saving.

#### Prognosis

Patients with DF in general have a good prognosis. Patients with severe DHF often progress to DSS with high mortality.

#### Vaccination

By recommendation of WHO countries have to consider dengue vaccine only where epidemiology suggests a high burden of disease, for use in persons 9–45 years of age, complete recommendations may be found in WHO releases. There is no global vaccination for DENV available, since April 2018 WHO revised recommendations for endemic regions.

### Nipah-virus (NiV)

#### Taxonomy, species, morphology, genome, reproduction, pathogenesis, virulence, transmission

Nipah-virus is an emerging bat-borne virus, belongs to the Paramyxoviruses, a group of negative sense, single stranded RNA viruses. Paramyxoviruses traditionally have been associated with a group of viruses that have narrow host range and typically cause outbreaks of disease with low mortality rates [15]. Fruit bats (Pteropus species) transmit Nipah but the number of human-to-human transmission of Nipah virus has increased, e.g. in Bangladesh [28].

#### Epidemiology

The name Nipah comes from the Malayan Kampung Sungai, here the virus was isolated the first time [5]. NiV outbreaks occur in the southeast Asian region with nearly annual frequency with sever diseases and high mortality rates.

The first recognized outbreak of NiV was 1998 in Malaysia, where humans and pigs were infected, due to transportation of infected pigs the virus was also recognized in other Malaysian regions. The transmission of NiV during this time was from pig to human, with the termination of about 900.000 pigs the epidemic was stemmed [1]. Australia, Bangladesh, Cambodia, China, India, Indonesia, Madagascar, Taiwan and Thailand are countries with reported outbreak risk based on serological evidence or molecular detection in pteropus bats. Pteropus bats are indigenous in Bhutan, Brunei, China, Indonesia, Laos, Madagascar, Myanmar, Nepal, Philippines, Singapore, Taiwan, Thailand and Vietnam.

#### Clinical manifestation

Incubation period ranges from 3 to 31 days with an average of 7 days.

Most patients present with acute encephalitis, fever, headaches and alteration of consciousness. During the epidemics in Malaysia patients also presented with a flue-like syndrome. According to the World Health Organization (WHO) the mortality rate is estimated at 40–75%, whereby variations during outbreaks can occur, depending on local capabilities for clinical management. For example, the mortality rate for the last outbreak in Kerala, India was 86%.

During the epidemics in Malaysia after an average of 10 days death occured. After several weeks, some of the patients suffered from a relapse.

#### Differential diagnosis

Other encephalitis triggering pathogens such as Japanese encephalitis, herpes-simplex-virus type 1- encephalitis, Dengue-Virus.

#### Diagnosis

CSF examination is crucial. For specific diagnosis virus isolation, electron microscopy, immune-electron microscopy, immunohistochemistry, serology and PCR can be used. Notice, only special laboratories (Biosafety Level 4- BSL4) are available to handle NiV. The specific detection can be made with CSF, serum, blood as well as swabs. For surveillance and diagnostic purposes three tests were developed not requiring BSL4: ELISA (enzyme-linked-immunoabsorbant assay), liquid protein array multiplex test and a pseudo type virus (different pseudo types carrying F and G proteins have been develop detecting virus-specific antibodies).

Neuroradiology: Magnetic resonance imaging (MRI) doesn’t distinguish other viral encephalitides. Abnormalities seen in MRI are multiple, small, asymmetric, focal lesions in the subcortical and deep white matter without surrounding oedema. Brainstem involvement is common.

#### Treatment

There is no specific treatment available. Specialized neurological intensive care management is essential for survival and prognosis. Ribavirin, which inhibits replication of Hendra Virus in vitro, was used during the outbreak in Malaysia, empirically. Recent animal models found that Ribavirin inhibits the viral spread in vitro but not in vivo. In the animal model Ribavirin delayed death from viral disease in NiV-infected animals for approximately for 5 days [9].

#### Prognosis

In vivo studies have shown, that Ribavarin is maybe effective to reduce mortality. Of patients who survive NiV encephalitis, 20% have residual neurological deficits, including cognitive difficulties, tetraparesis, cerebellar signs, nerve palsies and clinical depression.

#### Vaccinations

There is no commercial vaccine available.

### Japanese-encephalitis- B-virus (JEV)

#### Taxonomy, species, morphology, genome, reproduction, pathogenesis, virulence, transmission

JEV belongs to Flaviviridae with a single stranded RNA. JEV is transmitted by Culex mosquitos, commonly *Culex tritaeniorhynchus*, which breed in rice paddy fields and other water sources. Normally it’s an enzootic cycle, humans are the dead-end host for the virus. After infection, the virus replicates in the lymphoid tissue. From there a viremic spread and invasion into the CNS takes place. Inflammation occurs with focal necrosis of the nerve cells, neuroglia and perivascular lymphocytic infiltrates.

#### Epidemiology

JEV is endemic in South and South-east Asia. Less than 1% of individuals develop clinical features after incubation, about 20% of symptomatic patients develop a fatal encephalitis.

#### Clinical manifestation

The incubation period is about 5–15 days. Clinically patients may present with a flue-like illness. This may be followed by focal neurological signs, acute flaccid paralysis and subsequently severe life threatening encephalitis. Often there are significant neurological sequelae in patients surviving JEV encephalitis or acute flaccid paralysis.

#### Differential diagnosis

Other arthropod-born- virus induced diseases with the same geographic distribution of mosquitos like Chikungunya fever [7, 18].

#### Diagnosis

CSF examination is essential. A rapid test for the detection of IgM antibodies is available, with which serum and plasma can be tested. In the further course, increasing IgG titers can be detected. Neuroradiological examination may show involvement of thalamus and basal ganglia.

#### Treatment

There is no antiviral medication. Specialized neurological intensive care medicines is crucial.

#### Prognosis

Approximately 50% of the severe cases are lethal, 44% with mild or moderate course have a full recovery, 31% having persistent and severe neurologic sequelae, including chronic seizures, motor dysfunction and neuropsychiatric symptoms.

#### Vaccination

A vaccination against JEV is available, it is recommended for people living in or travelling to endemic regions. For Europe an inactivated vaccine i.m. is approved, primary immunization consists of two doses 28 days apart. The European Medicine Agency recommends a booster for exposed adults within the first year.

The inactivated mouse brain-derived vaccine is replaced now by cell culture-based vaccines, four types of cell culture-based vaccines are available. A live attenuated vaccine (SA 14–14-2 strain), mostly common in China, where the 1st dose is given subcutaneously at 8 months, followed by a booster dose at the age of 2 years. An inactivated vero cell-derived alum-adjuvanted vaccine (SA 14–14-2 strain), two intramuscular doses 4 weeks apart, booster is recommended after 1 year. The third one is the inactivated vero cell-derived vaccine (Beijing-1 strain), with primary immunization of three doses 4 weeks apart. The live chimeric vaccine (with yellow fever 17D as backbone), only a single dose is recommended, the need for booster have not been determined yet.

### Borna disease virus (BDV)

#### Taxonomy, species, morphology, genome, reproduction, pathogenesis, virulence, transmission

BDV is a member of the family *Bornaviridae* with a negative single-stranded nonsegmented RNA. The most likely natural route of entry is nerve endings in the nasal and pharyngeal nucosa. Infectious dose for natural infection is unknown, as well as transmission from infected animals to humans. Vertical transmission is confirmed, for animals and humans.

#### Epidemiology

In the 1700s borna disease was first described in European veterinary textbooks as a disease of farm horses with names like hot-headed disease (German: Hitzige Kopfkrankheit) or epidemic encephalomyelitis (German: Seuchenhafte Gehirn-Rückenmarksentzündung). The name borna disease was given after occurrence of major outbreaks in 1894–1896 in the district around Borna in Saxony, Germany.

BDV was the first member of the new family *Bornaviridae* in the order *Mononegavirales*, during the first decade of the twenty-first century a novel BDV, now known as avian bornavirus (ABV), was identified in parrots with proventricular dilatation disease. As known in veterinary medicine since many decades, recently bornavirus has been identified a causative pathogen of fatal encephalitis in humans. Between 2011 and 2013 three breeders of variegated squirrels (*Scirus variegatoides*) had encephalitis with similar signs and symptoms with lethal outcome two to 4 months after onset. Phylogenetic analysis revealed that the virus forms a lineage separate from the known bornavirus species, tentatively it’s named variegated squirrel 1 bornavirus (VSBV-1) [13].

#### Clinical manifestation

For humans with Borna virus infection no exact incubation time is specified. Animal studies have shown symptoms of disease after two to 3 weeks.

Neurological signs can be complex and variable, the variety of CNS infection can result in comparable clinical picture.

The recent published human cases had similar symptoms. All presented with an encephalitis, fever, qualitative and quantitative unconsciousness with initial cerebellar symptoms, myoclonic twitches and cranial nerve paresis [13].

#### Differential diagnosis

Tick-borne encephalitis, rabies, botulism, in regions with arthropod-borne flavivirus must be considered.

#### Diagnosis

CSF examination is crucial. Diagnosis can be confirmed with BDV-specific antibodies in serum and CSF or BDV-specific antigens, RNA detection in blood or CSF. Western blot analysis, ELISA and indirect immunofluorescence assay (IFA) have been established as serological test. IFA is considered as the most reliable method, with high sensitivity and specificity.

#### Treatment

There is no specific treatment available. For survival special neurological intensive care medicine is essential.

#### Vaccination

There is no vaccination available. As the vaccination for animals is not reliable, no further investigations for human vaccines are done at the moment.

### West-Nile-virus (WNV)

#### Taxonomy, species, morphology, genome, reproduction, pathogenesis, virulence, transmission

WNV belongs to the genus Flavivirus and is transmitted by culex mosquitos in an enzootic cycle. WNV contains ss-RNA that encodes the capsid(C), envelope (E), premembrane (prM) proteins as well as seven non-structural proteins. Several genetic lineages from WNV exist, lineage 1 and 2 are responsible for the major epidemics in humans. Replication takes place at the site of inoculation, thereafter the virus spreads to lymph nodes and bloodstream. CNS viral penetration appears to follow stimulation of toll-like receptors and increased levels of tumour necrosis factor- α (TNF- α), TNF- α increases permeability of the blood-brain-barrier. WNV directly infects neurons, particular in deep nuclei and grey matter of the brainstem and spinal cord.

#### Epidemiology

The first isolation of WNV was in 1937 in the West Nile district of Uganda. Historically there were sporadic outbreaks worldwide. Since the 1999 there were large number of outbreaks in North America, Southern and Eastern Europe, where smaller outbreaks occur annually.

#### Clinical manifestation

Most WNV infections are subclinical, apparently asymptomatic in about 80% [5].

Incubation time is 2–14 days, often West-Nile fever is self-limited. Classical symptoms of West-Nile fever are acute onset of fever, headache, fatigue, malaise, muscle pain and weakness. Rare occasions are gastrointestinal symptoms and a transient macular rash on the trunk and extremities. Some patients develop hepatitis, pancreatitis, myocarditis, rhabdomolysis, orchitis and ocular manifestations. Approximately < 1% develop neurological complications such as meningitis or encephalitis. WNV infection of spinal motor neurons (anterior horn cells) causes acute, asymmetric flaccid paralysis (AFP) similar to poliomyelitis. AFP occurs in about 13% of patients with neurological impairments [5]. The incidence of meningoencephalitis is higher in the older age group.

#### Differential diagnosis

Other arthropod-born- virus induced diseases with the same geographic distribution of mosquitos like chikungunya fever, dengue fever.

#### Diagnosis

CSF examination is crucial. Patients with neuroinvasive diseases generally have lymphocytic pleocytosis in CSF, neutrophils could be predominate in the early stage of disease. Laboratory diagnostics from CSF and blood samples include RT-PCR and IgM/ IgG analysis.

MRI is frequently normal but signal abnormalities in the basal ganglia, thalamus and brain stem can be seen in encephalitis patients, signal abnormalities in the anterior spinal cord in patients with poliomyelitis like syndrome.

#### Treatment

There is no specific treatment for WNV infection. Specialized neurological intensive care medicine is crucial for survival. Patients with severe meningitis often require pain control, antiemetic therapy and rehydration for associated nausea and vomiting. With severe encephalitis syndromes patients should be observed for development of elevated intracranial pressure and seizures. With paralysis or encephalitis patients should be monitored for inability to protect airway. Acute neuromuscular respiratory failure may develop rapidly, particularly ventilatory support may be required.

Individual patients have been reported where Ribavarin, interferon-α, WNV-specific immunoglobulin and antisense gene-targeted compounds have been considered but no clinical trails have been completed.

#### Vaccination

No human vaccination is available. Four veterinary vaccinations are used.

### Zika virus (ZIKV)

#### Taxonomy, species, morphology, genome, reproduction, pathogenesis, virulence, transmission

Zika virus belongs to the flaviviruses, it’s a positive single-stranded-enveloped RNA virus. The genome of ZIKV includes two noncoding regions and one region encoding a polyprotein that is cleaved into ten proteins including capsid, membrane precursor and envelope and seven non-structural proteins. ZIKV gains entry into host cells through endocytosis initiated upon interaction of virus envelope glycoprotein with cell surface receptors. *Aedes* mosquitos transmit ZIKV, mainly *Aedes aegypti*, it can also be transmitted from mother to foetus, through sexual contact, transfusion of blood and blood products and organ transplantation [2, 11, 16].

#### Epidemiology

In 1947 ZIKV was discovered in the Zika forest in Uganda, Africa. The first larger outbreak was reported in 2007 on Yap island, Micronesia after which the virus quickly spread to countries in Southeast Asia (French Polynesia in 2013, Cook Island and Easter Island in 2014), between 2015 and 2017 there was a dramatic increase of ZIKV cases in South America, that the World Health Organization announced a health emergency between 02/2016–11/2016.

#### Clinical manifestation

Estimated incubation time is three to 14 days. Generally the symptoms are mild with fever, rash, conjunctivitis, muscle and joint pain, malaise or headache. Most infected people do not develop symptoms. Neurological complications such as Guillian-Barré-like syndrome, neuropathy and myelitis can occur in adults as well as in children.

ZIKV can cause congenital syndromes in pregnancy. Meanwhile a causal context between ZIKV infection in pregnant women and subsequent malformations, such as microcephaly and severe brain damage has been confirmed. The virus passes through the placental barrier to infect foetal neural cells, to replicate and to induce severe neurological complications [19].

#### Differential diagnosis

Most flaviviruses with the same geographic region like DENV and CHIKV.

#### Diagnosis

Several diagnostic tools are available including Real-Time RT-PCR (rRT-PCR) assay and MAC-ELISA. RNA NAT should be performed with serum and urine specimens.

During the first week of illness ZIKV-specific IgM and neutralizing antibodies develop, IgM levels are variable, but generally positive near the 4th day of onset of symptoms.

IgM Antibody Capture Enzyme-linked immunosorbent Assay (MAC-ELISA) is used for qualitative detection of IgM antibodies in serum or CSF.

#### Treatment

As there is no specific antiviral therapy available, treatment is symptomatic.

#### Vaccination

No vaccine is available, protection against mosquito bites is the key prevention.

### Chikungunya-virus (CHIKV)

#### Taxonomy, species, morphology, genome, reproduction, pathogenesis, virulence, transmission

Chikungunya Virus also belongs to arthropod-borne viruses (family *Togaviridae*, genus *Alphavirus*), which causes endemic infections. CHIKV is transmitted by *Ae. aegypti* and *Ae. albopictus*, despite this CHIKV can be transmitted from the viremic mother to child during birth. Worldwide three phylogenetic CHIKV lineages with distinct antigenic characteristics are known, two major lineages circulate in Africa, the third one is present in Asia. Transmitting a specific variant of the African lineage *Ae. albopictus* is considered to have a high competence. The virus has a single amino acid change from alanine to valine, which leads to a better adaption of the virus in *Ae. albopictus* [12]*.*

#### Epidemiology

First isolation from humans and mosquitos were during an outbreak at the border region between Mozambique and Tanzania in 1952. In 2013 CHIKV was introduced into South America via the Caribbean, since then over 300,000 cases of infection and over 130 deaths have been reported [10].

CHIKV circulated in France in 2010, 2014 and 2017, in Italy with two major outbreaks in 2007 and 2017. Isolated CHIKV strains in Europa belong to the African lineage. By now *Ae. albopictus* has infected more than 25 European countries, highest abundances in Italy. In the past one considered that the area north of the Alps is unsuitable for establishment of *Ae. albopictus* but even in this region *Ae. albopictus* is overwintering (including local expansion of population and detection of larvae already in spring).

#### Clinical manifestation

CHIKV manifests high fever, headache, maculopapular rash and painful arthralgia with a typical incubation time of 3–7 days. Other symptoms could be retro-orbital pain. Myocarditis and haemorrhage, mostly the patients recover within 1–2 weeks, but joint pain may persist for several months to years.

During the outbreak on Réunion Island, 2005–2006, 12% of patients had neurological signs including quantitative and qualitative alteration of consciousness, focal neurological signs such as cranial nerve deficits, seizures, hemi/paraparesis and involuntary movements [27].

### Differential diagnosis

#### Diagnosis

Diagnosis is established by detecting CHIKV RNA with RT-PCR or virus serology during the first 5 days following onset of systems with high sensivity and specifity. A negative PCR initiate a prompt serological testing with enzyme-linked immunosorbent assay (ELISA) or indirect fluorescent antibody (IFA). CHIKV can be isolated from CSF, plasma or detected by nucleic acid amplification during the initial fever. IgM can persist for months, therefore detection of IgM antibodies can be useful. For differential diagnosis one should keep other arthropod-borne-diseases with the same geographic distribution in mind.

#### Treatment

There is no antiviral treatment for CHIKV, treatment is symptomatic, including specialized neurological intensive care medicine.

#### Prognosis

Usually, the prognosis for Chikungunya infection is good. Mortality is higher in elderly patients and immunosuppressed patients.

#### Vaccination

There is no vaccination available.

## Conclusion

Neurologist have to be aware of the possible occurrence of the emerging and re-emerging viruses such as Dengue-virus, Nipah virus, Japanese-Encephalitis-B-virus, Borna Disease virus, West-Nile-virus, Zika virus and Chikungunya virus.

In some of those vaccination is available, in all severe cases of central nervous system involvement specialized neurological intensive care medicine is crucial for survival and prognosis.

## Abbreviations

BBB: Blood-brain-barrier
BDV: Borna disease virus
BSL4: Biosafety level 4
CHIKV: Chikungunya virus
CNS: Central nervous system
CSF: Cerebrospinal fluid
DENV: Dengue-virus
DF: Dengue fever
DHF: Dengue haemorrhagic fever
DSS: Dengue shock syndrome
ELISA: Enzyme-linked-immunoabsorbant assay
GBS: Guillain-Barré-Syndrome
IFA: Indirect immunofluorescence assay
JEV: Japanese-Encephalitis- B-virus
MRI: Magnetic resonance imaging
NiV: Nipah-virus
PCR: Polymerase chain reaction
RKI: Robert Koch Institute
WHO: World Health Organization
WNV: West-Nile-virus
ZIKV: Zika virus
